# Investigation on the Antifungal Ingredients of *Saccharothrix Yanglingensis* Hhs.015, an Antagonistic Endophytic Actinomycete Isolated from Cucumber Plant

**DOI:** 10.3390/molecules24203686

**Published:** 2019-10-13

**Authors:** Hua Wang, Runze Tian, Qizhen Tian, Xia Yan, Lili Huang, Zhiqin Ji

**Affiliations:** State Key Laboratory of Crop Stress Biology for Arid Areas and College of Plant Protection, Northwest A&F University, Yangling 712100, Shaanxi, China; wangh619@foxmail.com (H.W.); tianrz2009@163.com (R.T.); bwcxzsqx@163.com (Q.T.); luckyx@126.com (X.Y.)

**Keywords:** pentaene macrolides, ultrastructure, *Valsa mali*, antifungal activity

## Abstract

Apple tree canker infected by *Valsa mali* var. *mali* is a serious and widely distributed disease in China. *Saccharothrix yanglingensis* Hhs.015 is an endophytic actinomycete isolated from cucumber roots, and it has been proven that this strain is a promising biocontrol agent on apple tree canker in previous studies. The aim of this study was to elucidate the active ingredients in its metabolites. Two pentaene macrolides, WH01 and WH02, were isolated from strain Hhs.015, and their structures were elucidated based on the extensive spectroscopic analysis. WH01 and WH02 were identified as fungichromin and 1′-deoxyfungichromin, among which WH02 is a novel compound. These two compounds showed strong in vitro and in vivo antifungal activity against *V. mali*. By comparison of the structures of hyphae cells treated by pure compound and fermentation broth, it has been proven that pentaene macrolides are the main active ingredients in the metabolites of strain Hhs.015. This is the first report on the antifungal activity of fungichromin and its analogs on *V. mali*, and the 28-member pentaene macrolides were also firstly isolated from the genus of *Saccharothrix.*

## 1. Introduction

Natural products have been an important source of active ingredients for the discovery of novel fungicides [1]. Antibiotics are the most famous natural product in the history of fungicide development. In the 1960s and 1970s, a large number of registered fungicides belonged to antibiotics, such as blasticidin-S, kasugamycin, mildiomycin, polyoxin, and validamycin, and were registered in many countries. During this period, most active ingredients of commercialized antibiotics were produced from the *Streptomyces* genus. Consequently, the chances of finding new antibiotic ingredients from *Streptomyces* were gradually decreasing. As a new channel of producing antibiotics, non-*Streptomyces* actinomycetes attracted more attention from pharmaceutical and agro-chemical industries in recent years, and a large number of novel compounds were isolated from the genera of non-*Streptomyces* such as *Micromonospora* [2,3], *Nocardia* [4], *Actinomadura* [5], *Actinoplanes* [6], and *Saccharopolyspora* [7], respectively.

Apple tree canker infected by *Valsa mali* var. *mali* is a serious and widely distributed disease in China, and causes great economic losses to fruit farmers every year [8]. Currently, the primary treatment strategy is to apply chemical fungicides on the tree trunk after the canker lesion is scraped [9]. Although the disease could be effectively controlled by this procedure, the exposure of high-level toxic chemicals has become a great threat to farmer and environment. There is an urgent need for environmental friendly alternatives to address this problem. As a potent resource for biological control agents, screening antifungal ingredients from microorganisms has attracted more and more attention [10].

The genus *Saccharothrix* was firstly isolated from a soil sample in 1984 [11]. Up to now, several types of antibiotics including dithiolopyrrolone [12,13], anthracycline [14], and ammocidin [15] have been isolated from *Saccharothrix*. In the screening process of antibiotics, an endophytic actinomycete strain Hhs.015 was isolated from roots of cucumber in our laboratory in 2009 [16]. In the following years, the investigations on the taxonomy and antifungal activity of strain Hhs.015 have been thoroughly carried out. Based on polyphasic taxonomy and 16S rRNA sequence analysis, the strain was identified as a novel species and named as *Saccharothrix yanglingensis* Hhs.015 [17]. The fermentation broth of strain Hhs.015 exhibited strong antifungal activity against apple tree canker both in laboratory and field circumstances [18]. The germination of *V. mali* conidia could be effectively inhibited by the metabolites of Hhs.015, and the fungal mycelial became crimped while the hyphae tips were extremely ramified. Field trials showed that apple trees were protected from the infection of *V. mali* with treatment of Hhs.015, and its control efficacy was comparable to that of commercial chemical fungicide difenoconazole [19]. A newly published article revealed that Hhs.015 could produce an endo-type chitinase (Chi6769), and exhibited antifungal activity against *V. mali* via hydrolysis of chitin [20]. Further studies on antifungal mechanisms revealed that a novel protein elicitor, BAR11, was identified from Hhs.015, which could trigger defense responses in plants [21]. As a promising biocontrol agent, optimization of the fermentation process of Hhs.015 was also carried out, and the antimicrobial activity was increased by 20% at the optimum condition [22].

Although we have done a lot of research on the antifungal activity and mechanism of the fermentation broth previously, the antifungal ingredients in the metabolites of Hhs.15 still remain unclear [23]. It is urgent to identify the specific antimicrobial substances produced by the strain. In this study, two polyene macrolide antibiotics were isolated from the fermentation broth of *Saccharothrix yanglingensis* Hhs.015, and their structures were elucidated based on extensive spectrometry analysis. Here we report the isolation, structural elucidation, and antifungal activity of polyene macrolides produced by strain Hhs.015.

## 2. Results and Discussion

### 2.1. Isolation of Antifungal Polyene Macrolides

**Figure 1:**
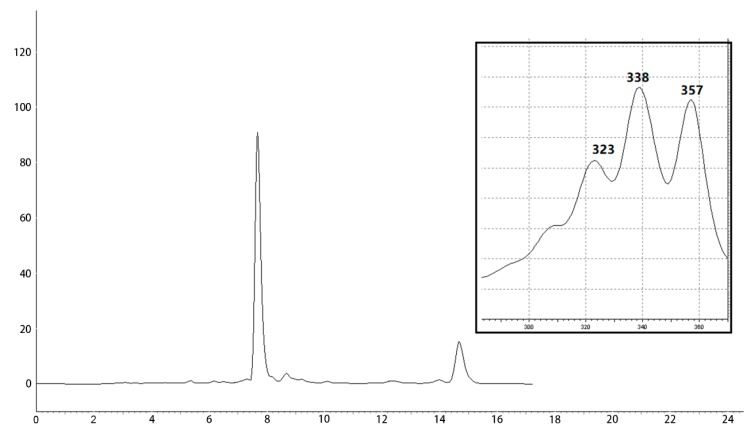
Chromatogram of antifungal fraction and UV spectrum of the main peak.

The isolation of the antifungal ingredients in the metabolites of strain Hhs.015 was carried out on the basis of bioassay. Seventeen fractions were obtained from the silica gel column chromatography, among which Fr.09, Fr.10 and Fr.11 exhibited antifungal activity against *V. mali*. HPLC analysis revealed the chemical composition of these three fractions was similar. The active fractions were combined and subjected to semipreparative HPLC for further purification. The chromatogram was monitored at 338 nm, and peaks 1 (WH01) and 2 (WH02) were observed at the retention times of 7.7 and 14.7 min, respectively (Figure 1). Their UV spectra are nearly identical, which implied that their structure was related, and the ratio of their peak areas was 5:1.

### 2.2. Structural Elucidation

**Figure 2:**
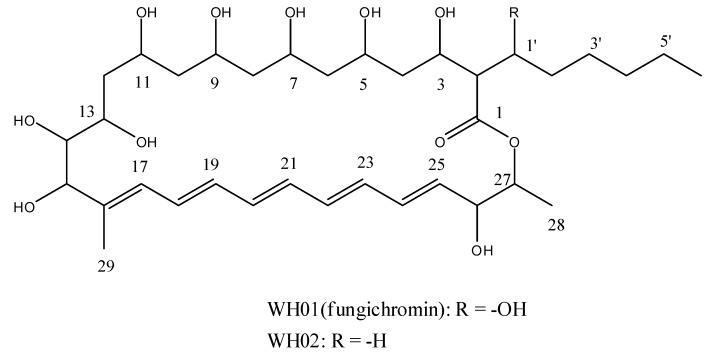
Chemical structures of WH01 and WH02.

The chemical structures of WH01 and WH02 were elucidated by HRMS, 1D, and 2D NMR analysis (Figure 2).

WH01 could be identified as fungichromin by comparing its NMR data with previous study [24].

**Figure 3:**
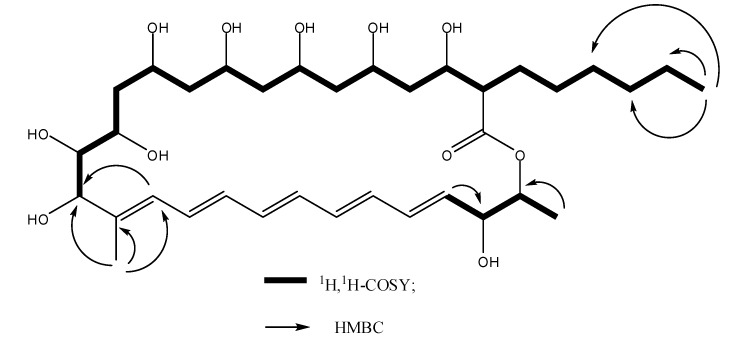
Key 2D NMR correlations of WH02.

WH02 was obtained as a yellow amorphous solid. The UV spectrum of WH02 exhibited the typical absorption bands of pentaene macrolides at 338, 357, and 323 nm. The molecular formula was established as C_35_H_58_O_11_ by HRMS (*m/z* 677.3830, [M + Na]^+^; calcd for C_35_H_58_O_11_Na, 677.3877). Based on the ^13^C-NMR, DEPT, and HSQC spectra of WH02, 35 carbon atoms were observed, which could be recognized as one quaternary, nine *sp* methine, eleven *sp^3^* methine, ten methylene, three methyl, and one carbonyl carbons, respectively. Except the deviation in chemical shifts of several carbon atoms, the ^1^H and ^13^C-NMR spectra of WH01 and WH02 are very similar, presumed that WH02 is a structural analog of fungichromin. The results of HRMS analysis revealed that fungichromin has one more oxygen atom than WH02. Meanwhile, the chemical shifts of C-2, C-1′, and C-2′ carbon atoms in the ^13^C-NMR spectrum of WH02 were dramatically shifted upfield compared to those of WH01, while other signals of carbon atoms of these two compounds are nearly identical. The cross peaks between H-2 (*δ* 2.48)/H-1′(*δ* 3.97) and H-2 (*δ* 2.48)/H_3_(*δ* 3.68) were observed in the ^1^H,^1^H-COSY spectrum of WH02, which indicated that the hexane side chain was substituted at C-2 of the 28 member macrolide moiety. The chemical shifts of H-1′of WH02 were shifted upfield obviously, which implied that the hydrogen atom in C-1′ of the hexane side chain was not substituted by the hydroxyl group. The substituted positions of two methyl groups and nine hydroxyl groups at the macrocyclic lactone ring were determined by comparing its chemical shifts of hydrogen and carbon atoms with those of fungichromin, as well as the correlation observed in the ^1^H, ^1^H-COSY and HMBC spectra (Figure 3). Thus, the chemical structure of WH02 could be identified as 1′-deoxyfungichromin. Detailed NMR and HRMS data of compound WH02 will be shown in the Supplementary Materials.

### 2.3. Antifungal Activity of Polyenes against V. mali

WH01 and WH02 could effectively inhibit the mycelial growth of *V. mali*, and their EC_50_ values were 5.24 and 4.92 mg/L (Table 1), respectively. The only structural difference between them is that WH01 has a hydroxyl group at the C-1′of the hexane side chain, whereas it is not present in WH02. It implied that the presence of the hydroxyl group substituted at the hexane side chain was not crucial to the antifungal activity of fungichromin.

**Figure 4:**
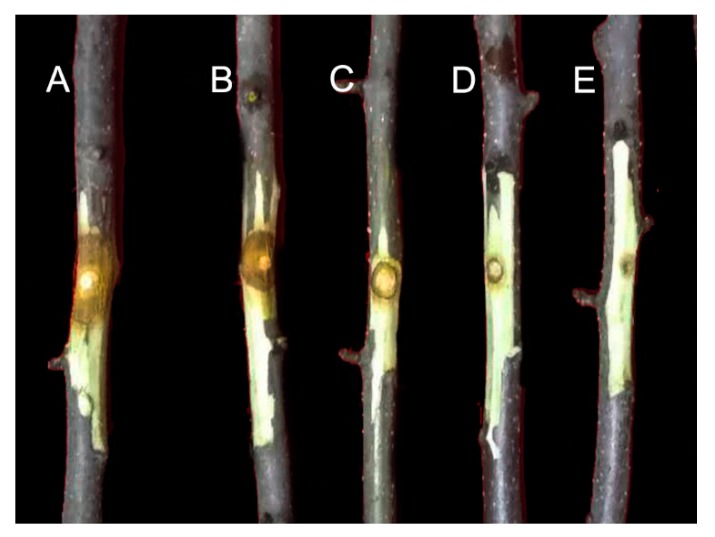
Protective effect of WH01 on apple tree canker infected by *V. mali.* (**A**) Negative control, (**B**) 250 mg/L, (**C**) 500 mg/L, (**D**) 1000 mg/L, and (**E**) 2000 mg/ml.

WH01, the main active component, was selected for further in vivo tests. The protective effect of WH01 on apple tree canker infected by *V. mali* was evaluated using detached twig assays. As shown in Figure 4 and Table 2, WH01 significantly inhibited the lesion expansion on twigs. After treatment for 7 days at 2000, 1000, 500, and 250 mg/L of WH01, the protective effects were 96.30%, 81.48%, 74.07%, and 44.44%, respectively.

### 2.4. Light Microscopy and Transmission Electron Microscopy Analyses

**Figure 5:**
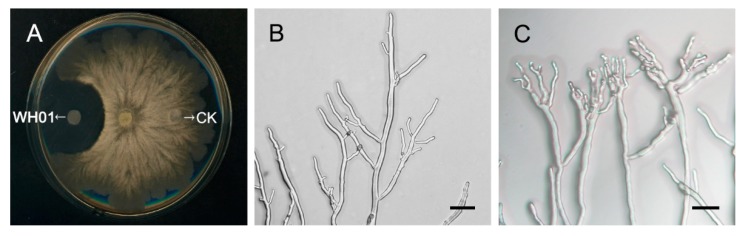
Inhibition effect of WH01 on *V. mali*. (**A**) Inhibition effect of WH01 and CK (compound-free filter paper) on hyphae of *Valsa mali*. WH01 could inhibit the continuous expansion of hyphae. (**B**) Morphology of hyphae collected from opposite of the dish, which were not affected by WH01 treatment. (**C**) Morphology of hyphae collected from the WH01-treated side. The swollen hyphae branched to form clusters with serious malformation. Bar = 50 μm.

WH01 could significantly inhibit the continuous expansion of hyphae comparing to CK, compound-free filter paper (Figure 5A). After treatment with WH01, the morphology of hyphae showed obvious anomalies. The swollen and branched apexes forming clusters were displayed under the treatment (Figure 5C). And the clusters contracted to be malformative. However, the hyphae under the CK showed the normal and smooth branches (Figure 5B).

**Figure 6:**
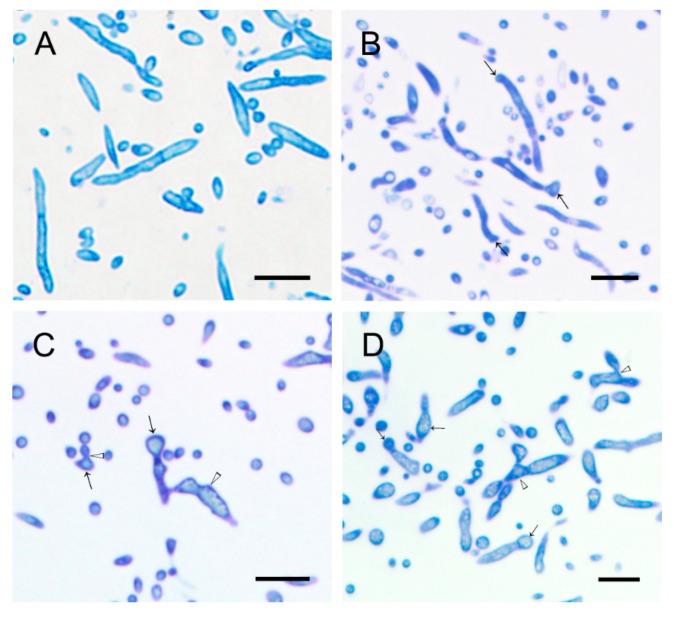
Morphology of hyphae of *V. mali* under different concentrations of WH01 treated at 48 h. (**A**) At 0 mg/L treatment, healthy hyphae were exhibited. (**B**) At 3 mg/L treatment, morphological differences were not significant. The swollen hyphae were exhibited. At (**C**) 6 mg/L and (**D**) 12 mg/L treatment, most hyphae were malformative. Bar = 10 μm. (“→”: swollen hypha, “△” malformation of hypha).

**Figure 7:**
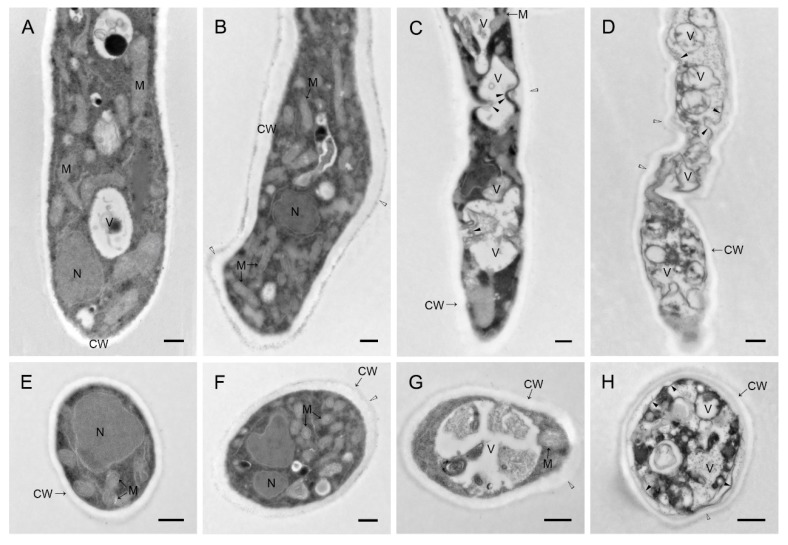
Cytology of hyphae of *V. mali* under different concentrations of WH01 treated at 48 h. (**A**) and (**E**) were the transverse and longitudinal sections of hyphae, respectively, under 0 mg/L treatment. (**B**) and (**F**) were the transverse and longitudinal sections of hyphae WH01 treatment, respectively, under 3 mg/L treatment. Mitochondrial contents in hyphae increased with malformation of hyphae. (**C**) and (**G**) were the transverse and longitudinal sections of hyphae, respectively, under 6 mg/L treatment. Malformation and vacuolization of hyphae with plasmolysis slightly was exhibited. (**D**) and (**H**) were the transverse and longitudinal sections of hyphae, respectively, under 12 mg/L treatment. The range of malformation, vacuolization, and plasmolysis of hyphae increased more. Bar = 500 nm. (CW: cell wall, N: nucleus, M: mitochondria, V: vacuole, “▲” (black triangle): plasmolysis, “△” (white triangle): malformation of hyphae)

Compared to the control group, WH01 obviously induced vacuolization of hyphae, and the degree of vacuolization was positively correlated with the treated concentration (Figure 6). Healthy hyphae had a uniformly thin cell wall and diverse intact organelles (Figure 7A,E). Hyphae under 3 mg/L treatment showed a thickening of the cell wall and increased mitochondria and the slight malformation on morphology (Figure 7B,F). Meanwhile, malformation and vacuolization of hyphae occurred in both 6 and 12 mg/L treatments (Figure 7C,G,D,H). Besides, organelles such as mitochondria were partially intact under 6 mg/L and degradation occurred in the cells. And it had been unable to observe a normal organelle in hyphae because more serious plasmolysis and vacuolization occurred in the cells under 12 mg/L treatment. The effects of WH01 on the microstructures of mycelia were similar to those treated by the fermentation broth of strain Hhs.015 previously [19]. It is reasonable to conclude that WH01 and WH02 are the main antifungal substances in strain Hhs.015.

In summary, strain Hhs.015 is a promising biocontrol agent on apple tree canker, and the results revealed that pentaene macrolides are the active ingredients in the metabolites of strain Hhs.015. To our best knowledge, this is the first report on the in vitro and in vivo antifungal activities of fungichromin and its analogs on *V. mali*, and the 28-member pentaene macrolides were also firstly isolated from the genus of *Saccharothrix.*

## 3. Materials and Methods

### 3.1. General Procedure

Nuclear magnetic resonance spectra were measured on a Bruker Avance III-500 NMR spectrometer (Bruker BioSpin Inc., Rheinstetten, Germany). Chemical shifts were referenced to residual solvent signals. The high-resolution mass spectrum was measured on a TOF/MS instrument (Agilent Technologies Co., Santa Clara, CA, USA). The final step for purification was performed on a HPLC apparatus equipped with a diode array detector (LC-6AD, Shimadzu Co., Kyoto, Japan) and a C_18_ column (SinoChrom ODS-BP, 250mm × 10mm, 10μm; Elite Co., Dalian, China). The silica gel for column chromatography was purchased from Haiyang Chemical Co., Ltd (Qingdao, China). The instruments, including light microscopy BX51/DP70 microscope (OLYMPUS, Tokyo, Japan), transmission electron microscopy HT-7700 (HITACHI, Tokyo, Japan), and microtome UC7 (LEICA, WetzlarGermany), were used to analyze the changes of hyphae under diverse condition.

### 3.2. Microorganisms and Culture Conditions

*S. yanglingensis* Hhs.015 and apple canker pathogen *Valsa mali* var. *mali* were provided by the Laboratory of Integrated Management of Plant Diseases, College of Plant Protection, Northwest A&F University, China.

SSY liquid medium was consisted of soybean powder 20 g, sucrose 10 g, soluble starch 5 g, yeast extract 2 g, protease peptone 2 g, NaCl 2g, CaCO_3_ 1 g, MgSO_4_•7H_2_O 0.5 g, KH_2_PO_4_ 0.5 g, and 1 L double distilled water [22].

Gause’s Synthetic Agar (GSA) medium was consisted of soluble starch 20 g, NaCl 0.5 g, KNO_3_ 0.5 g, K_2_HPO_4_ 0.5 g, MgSO_4_•7H_2_O 0.5 g, FeSO_4_•7H_2_O 0.01 g, agar 15 g, and 1 L double distilled water.

Potato Dextrose Agar (PDA) medium was consisted of potato 200 g, dextrose 20 g, agar 10 g, and 1 L double distilled water.

Strain Hhs.015 was kept on GSA medium at 4 °C. For spore production, the strain was transferred to GSA plates and incubated for 6 days in darkness at 28 °C. The produced spores were used for inoculation in the SSY liquid medium. Spore cakes (diameter 5 mm) of Hhs.015 were prepared to SSY medium. Each flask was inoculated with five spore cakes (250 mL flask containing 50 mL SSY medium). The flasks were incubated at 28 °C on a rotary shaker at 150 rpm for 3 days to generate seed culture liquid. Large scale fermentation was carried out in 250 mL flasks containing 80 mL SSY liquid medium. These flasks were inoculated with 10% (*v/v*) seed culture liquid, and incubated at 28 °C on a rotary shaker at 150 rpm for 6 days.

### 3.3. Extraction, Isolation, and Structural Elucidation of Antifungal Ingredients

The fermentation broth (12 L) of strain Hhs.015 was centrifuged at 6760 g (relative centrifugal force) for 10 minutes. The supernatants were collected and extracted with ethyl acetate (5 L) over three times, and the solvents were evaporated under vacuum. The sediments were extracted with methanol (5 L) under ultrasonic for 30 min over three times and concentrated in vacuo. The concentrated extracts of supernatants and sediments (12.6 g) were combined and subjected to a silica gel column chromatography. The column was eluted with the mixture of ethyl acetate and methanol at the ratio from 1/0 to 4/1 (*v/v*). The antifungal fractions were combined and subjected to semi-preparative HPLC for further purification on a C_18_ column and eluted with 80% methanol. Two peaks were collected based on the HPLC profiles, and these two fractions were evaporated under vacuum to afford compounds WH01 and WH02. The structural elucidation of the obtained pure compounds was performed using NMR and mass spectrometry analysis.

WH01. Yield 68 mg; yellow amorphous solid; HRMS *m/z* 693.3816, [M + Na]^+^ (calcd for C_35_H_58_O_12_Na, 693.3826); ^1^H and ^13^C-NMR (Table 3).

WH02. Yield 12 mg; yellow amorphous solid; HRMS *m/z* 677.3830, [M + Na]^+^ (calcd for C_35_H_58_O_11_Na, 677.3877); ^1^H and ^13^C-NMR (Table 3).

### 3.4. In Vitro Antifungal Activities of WH01 and WH02 against V. mali

The antifungal activity of the obtained compounds WH01 and WH02 against *V. mali* was evaluated using mycelial growth method [25]. The antibiotics were dissolved with dimethylsulfoxide (DMSO) and diluted to appropriate concentration by melted PDA medium. Then, the mixture was transferred to the Petri dish, left to solidify at room temperature, and then inoculated with the agar discs of tested fungi. 1,2-Benzisothiazolin-3-One were used as the positive control. Aliquots of DMSO were used as the negative control. Then, the microorganisms were incubated at 25 °C for 48 h, and the inhibitory effect was calculated through colony diameter. Each treatment was performed with three replicates.

### 3.5. In Vivo Antifungal Activity of WH01 against V. mali

The in vivo antifungal activity of the obtained polyene macrolide antibiotics against *V. mali* was evaluated using detached twig assays in laboratory [19]. The healthy twigs from *Malus domestica* cv. Fuji were treated as previously described in the literature [26]. The methanol solution of tested samples was uniformly daubed onto the surface of the treated segment. After air drying in room temperature, mycelial blocks of *V. mali* were inoculated onto the treated surface. The treated apple tree twigs were placed in a moisture-controlled chamber at 25 °C. Methanol was used as a negative control. The sizes of lesions were recorded at 7 days after the inoculation. Each treatment was performed with five replications.

### 3.6. Light Microscopy

The inhibition effect of WH01 on hyphae of *V. mali* was designed according to Liu and Fan et al. [27] descriptions. The *V. mali* stored at −80 °C were cultured in PDA medium for 48 hours, and then transferred to the center of the other PDA solid medium. Two filter papers were immersed in the WH01 and methanol (CK), respectively, and, after air drying in room temperature, were put on opposite sides in the same PDA medium as transferring *V. mali* to continue culturing for 48 hours, to observe the inhibition effect of WH01 on *V. mali.* The samples of hyphae were collected from the sites treated with WH01 and its opposite site treated with methanol in dishes. Then the samples were observed using light microscopy.

### 3.7. Transmission Electron Microscope

The *V. mali* stored at −80 °C were cultured in PDA medium for 48 hours, and then transferred to another PDA medium containing WH01 with the concentrations of 0 mg/L, 3 mg/L, 6 01/L, and 12 mg/L, respectively, to continue culturing for 48 hours, which were used to study the histological and cytological changes of hyphae under different treatments. The method of transmission electron microscopy was followed as Kang and Buchenauer described [28]. The semithin sections were stained with toluidine blue and observed by BX51/DP70 microscope (OLYMPUS, Tokyo, Japan). And the ultrathin sections were stained with uranyl acetate and lead citrate, then observed by HT-7700 transmission electron microscope (HITACHI, Tokyo, Japan) at 80.0-kV voltage.

### 3.8. Statistical Analysis

Data analysis was conducted using the SPSS11.0 (SPSS Inc., Chicago, IL, USA). Regression analyses were undertaken between the concentrations of tested compounds and the inhibition rate of the pathogen mycelial growth, with correlation coefficient (R^2^) and the half maximal effective concentration (EC_50_) calculated.

## Figures and Tables

**Table 1:** In vitro antifungal activity of polyenes against *Valsa mali*.

Compounds	EC50 (95% Confidence Interval) mg/L	Toxicity Regression Equation (Y = a + bx)	*R^2^*
WH01	5.24 (4.36~6.21)	Y = −1.52 + 2.12x	0.99
WH02	4.92(4.07~5.87)	Y = −1.42 + 2.05x	0.99
1,2-Benzisothiazolin-3-One	8.85(7.09~11.33)	Y = −2.26 + 2.42x	0.98

**Table 2:** In vivo antifungal activity of **WH01** on *V. mali*.

Chemicals	mg/L	Protective Effect (%)
**WH01**	250	44.44%
	500	74.07%
	1000	81.48%
	2000	96.30%

**Table 3:** ^1^H and ^13^C-NMR shift assignments of WH01 and WH02 in DMSO-*d_6_*.

	WH01,(Fungichromin)		WH02	
Position	δ C	δ H	δ C	δ H
1	170.6		172.6	
2	58.2	2.48(m)	51.9	2.25(ddd, J = 11.0, 8.0, 3.5 Hz)
3	69.1	3.68(m)	70.3	3.62(m)
4	39.6	1.37(m), 1.56(m)	39.2	1.37(m), 1.56(m)
5	69.8	3.89(m)	69.2	3.87(m)
6	43.4	1.38(m)	43.0	1.31(m), 1.49(m)
7	69.6	3.77(m)	69.0	3.68(m)
8	43.5	1.31(m), 1.49(m)	43.3	1.31(m), 1.49(m)
9	68.8	3.87(m)	68.7	3.87(m)
10	42.4	1.32(m), 1.47(m)	42.1	1.32(m), 1.47(m)
11	70.1	3.88(m)	70.1	3.88(m)
12	38.2	1.37(m), 1.56(m)	38.1	1.37(m), 1.56(m)
13	67.9	3.14(m)	67.9	3.14(m)
14	76.1	3.49(d, J = 9.0 Hz )	76.0	3.49(d, J = 9.0 Hz)
15	77.6	3.69(d, J =9.0 Hz)	77.4	3.69(dd, J=9.0, 2.5 Hz)
16	138.3		138.2	
17	126.7	5.94(d, J = 11.0 Hz)	126.5	5.94(m)
18	127.4	6.45(dd, J = 15.0, 11.0 Hz)	127.4	6.45(dd, J = 15.0, 11.0 Hz)
19	130.8	6.26(m)	131.0	6.27(m)
20	128.6	6.26(m)	128.6	6.26(m)
21	132.6	6.35(m)	132.2	6.36(m)
22	131.8	6.26(m)	131.9	6.28(m)
23	132.7	6.35(m)	132.4	6.32(m)
24	132.8	6.35(m)	132.4	6.32(m)
25	134.6	6.02(dd, J = 15.0, 4.0 Hz)	134.4	5.95(m)
26	70.7	3.96(m)	70.9	3.96(dd, J = 9.0, 5.0 Hz)
27	72.7	4.63(m)	72.4	4.63(m)
28	17.3	1.19(d, J = 6.5)	16.9	1.22(d, J = 6.0Hz)
29	11.2	1.69(s)	11.2	1.69(s)
1′	70.6	3.97(m)	28.5	1.44(m),1.65(m)
2′	33.7	1.27(m), 1.36(m)	25.9	1.14(m),1.21(m)
3′	24.1	1.25(m), 1.43(m)	27.9	1.23(m)
4′	30.8	1.23(m)	30.4	1.22(m)
5′	21.6	1.25(m)	21.3	1.25(m)
6′	13.5	0.86(t, J = 7.5Hz)	13.3	0.85(t, J = 7.5Hz)

## Supplementary Materials

Figure **S1**: ^1^H-NMR spectrum of WH02 (500 MHz, DMSO-*d6*), **Figure S2**:^13^C-NMR spectrum of WH02 (125 MHz, DMSO-*d6*), **Figure S3**: DEPT spectrum of WH02 (125 MHz, DMSO-*d6*), **Figure S4**: ^1^H, ^1^H-COSY spectrum of WH02 (600 MHz, DMSO-*d6*), **Figure S5**: HSQC spectrum of WH02 (600 MHz, DMSO-*d6*), **Figure S6**: HMBC spectrum of WH02 (600 MHz, DMSO-*d6*), **Figure S7**: NOESY spectrum of WH02 (600 MHz, DMSO-*d6*), **Figure S8**: HRMS spectrum of WH02.

## Abbreviations

16S rRNA	16S ribosomal RNA
Fr.	Fragment
HPLC	High Performance Liquid Chromatography
UV	Ultraviolet
HRMS	High Resolution Mass Spectrometer
NMR	Nuclear magnetic resonance
DEPT	Distortionless Enhancement by Polarization Transfer
HSQC	Heteronuclear singular quantum correlation
COSY	Correlation Spectroscopy
HMBC	1H detected heteronuclear multiple bond correlation
NOESY	Nuclear overhauser effect spectroscopy
GSA	Gause’s Synthetic Agar medium
PDA	Potato Dextrose Agar medium
DMSO	Dimethylsulfoxide
