# Supplementary material for: Investigation on the Antifungal Ingredients of Saccharothrix Yanglingensis Hhs.015, an Antagonistic Endophytic Actinomycete Isolated from Cucumber Plant
Source: Molecules. 2019 Oct 13;24(20):3686. doi: 10.3390/molecules24203686 (PMC6833113; doi:10.3390/molecules24203686)
Supplement: Supplementary file 1 [file molecules-24-03686-s001.pdf]

molecules

Supplementary Materials

Investigation on the Antifungal Ingredients of *Saccharothrix Yanglingensis* Hhs.015, an Antagonistic Endophytic Actinomycete Isolated from Cucumber Plant

Hua Wang, Runze Tian, Qizhen Tian, Xia Yan, Lili Huang * and Zhiqin Ji *

State Key Laboratory of Crop Stress Biology for Arid Areas and College of Plant Protection, Northwest A&F University, Yangling, Shaanxi 712100, China; wangh619@foxmail.com (H.W.), tianrz2009@163.com (R.T.), bwcxzsqx@163.com (Q.T.), luckyx@126.com (X.Y.)

* Correspondence: jizhiqin@nwsuaf.edu.cn (Z.J.); huanglili@nwsuaf.edu.cn (L.H.); Tel.: +86-15309203829 (Z.J.); +86-029-87091312 (L.H.)

Received: 16 September 2019; Accepted: 11 October 2019; Published: 12 October 2019
Academic Editor: Isabel C.F.R. Ferreira

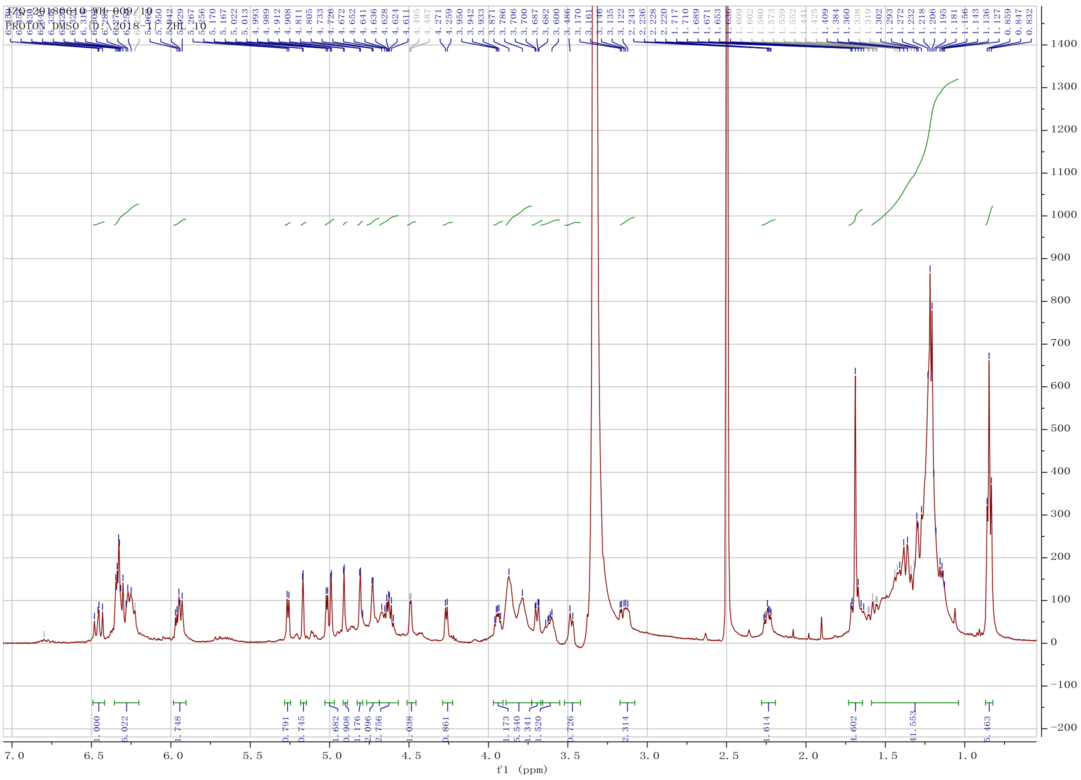

Figure 1. ^{1}H NMR spectrum of WH02 (500 MHz, DMSO-d_6).

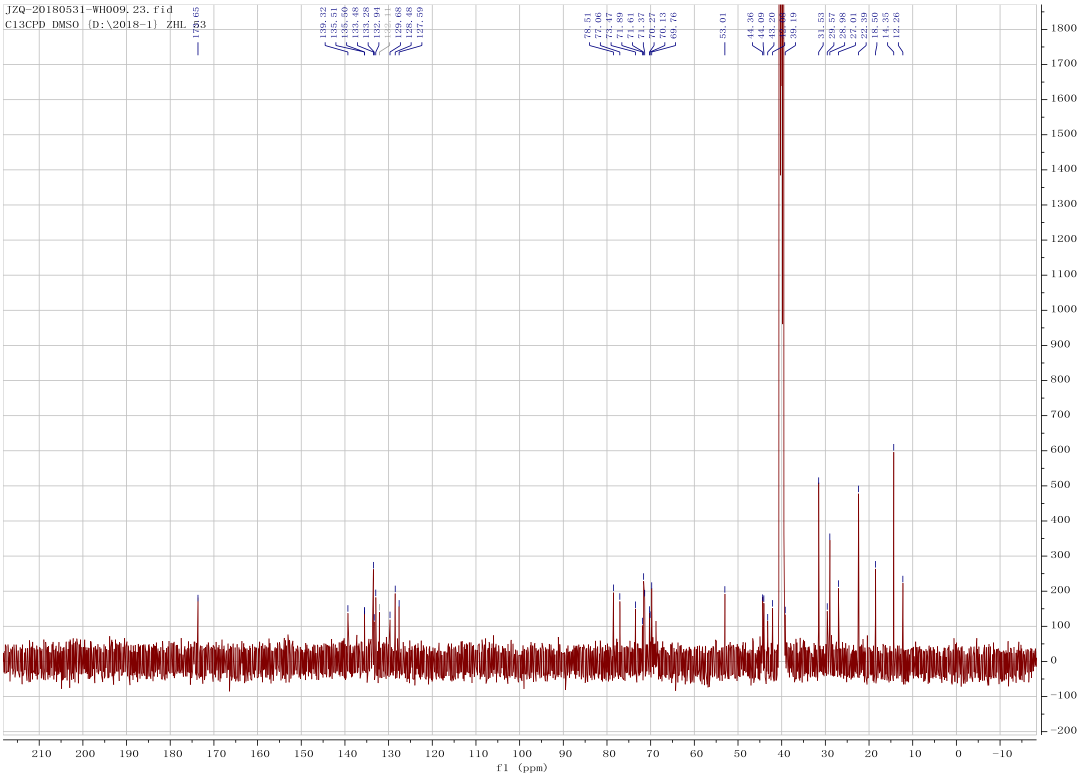

Figure 2. ^{13}C NMR spectrum of WH02 (125 MHz, DMSO-d_6).

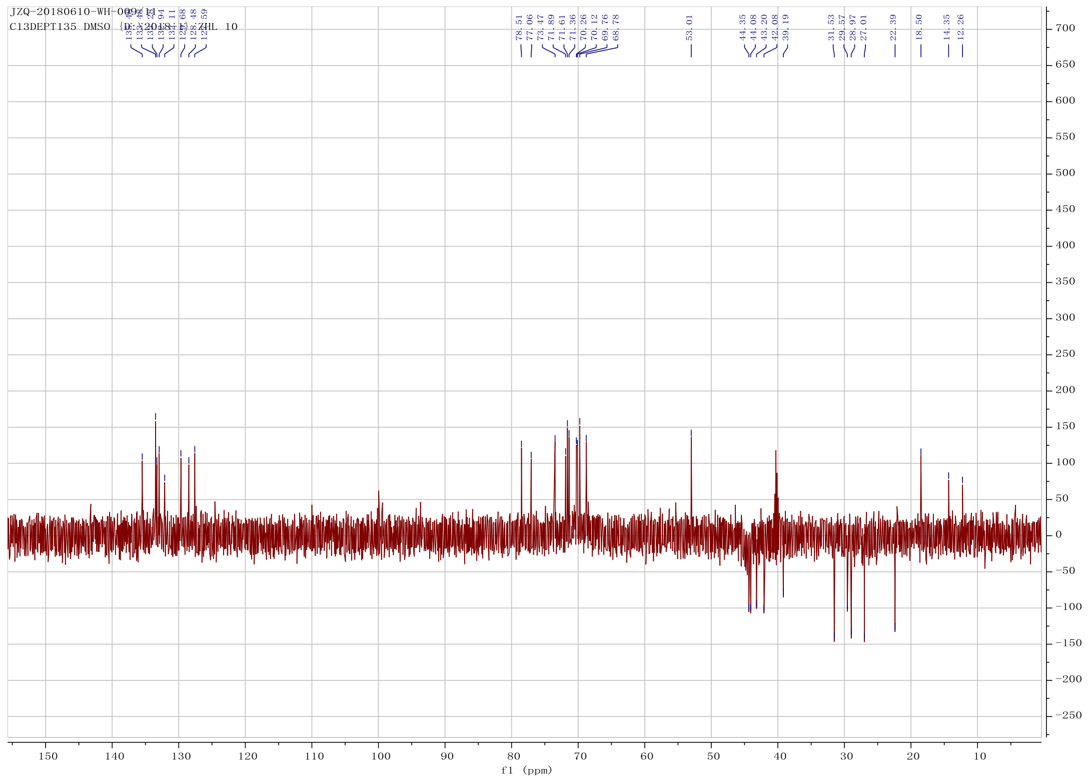

Figure 3. DEPT spectrum of WH02 (125 MHz, DMSO-d_6).

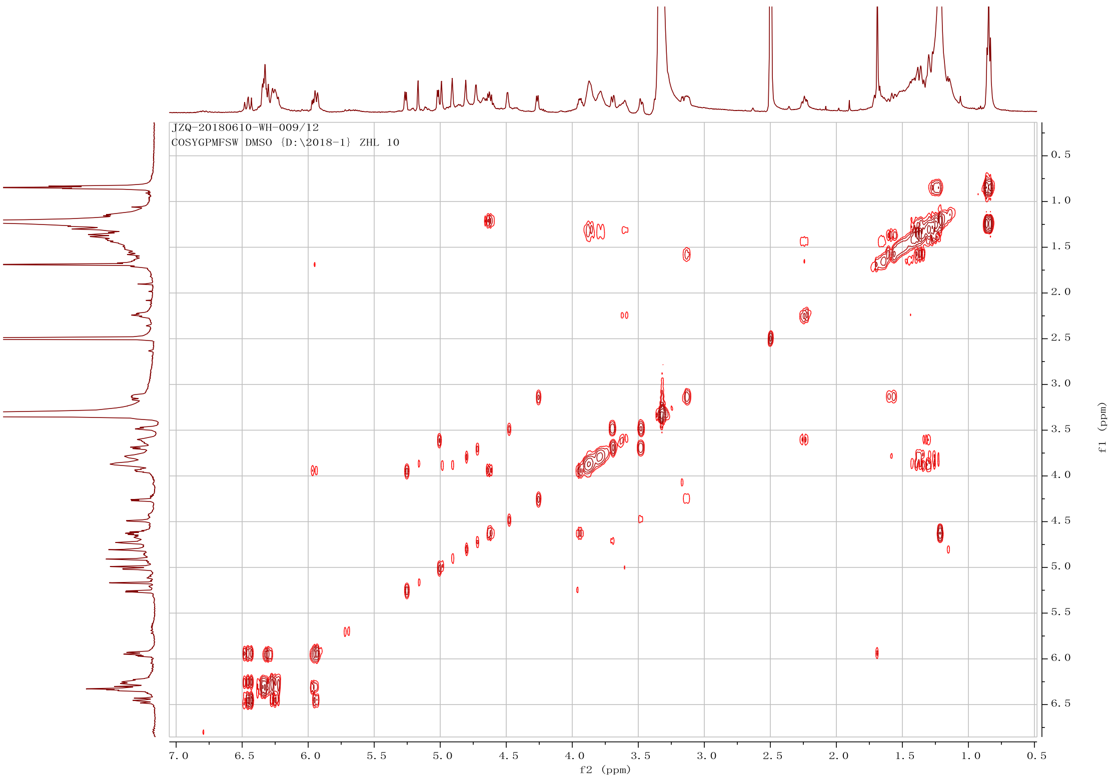

Figure 4. ^{1}H,^{1}H-COSY spectrum of WH02 (600 MHz, DMSO-d_6).

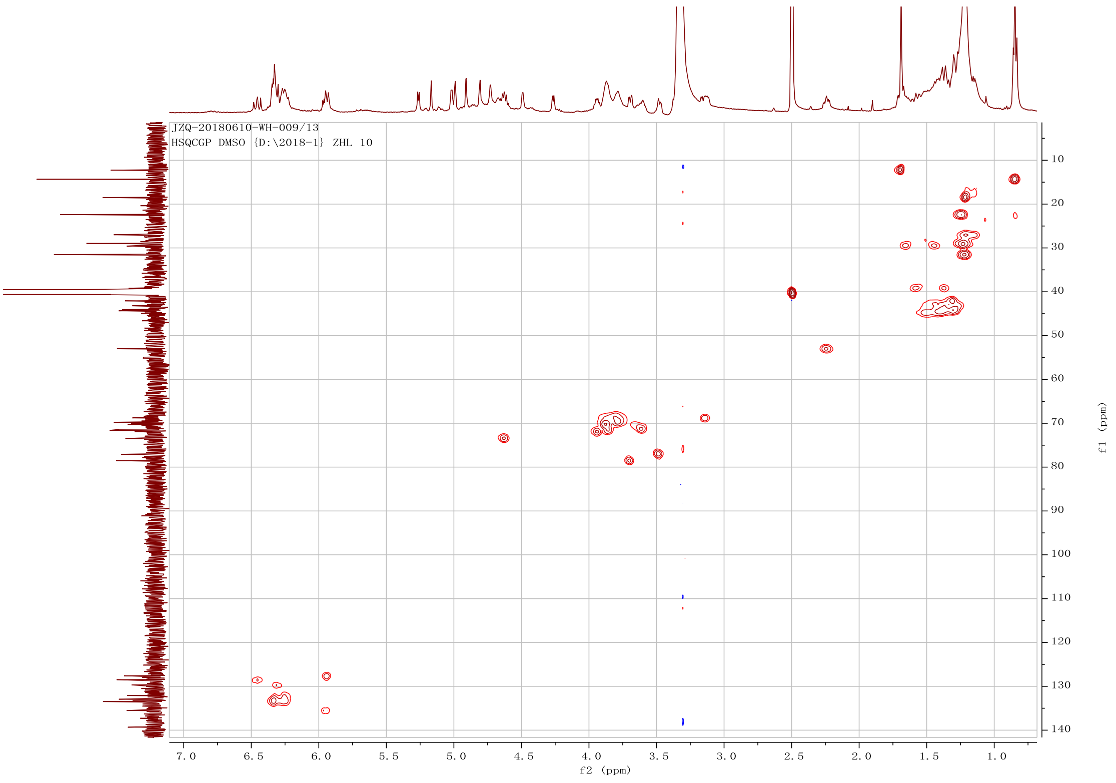

Figure 5. HSQC spectrum of WH02 (600 MHz, DMSO-d_6).

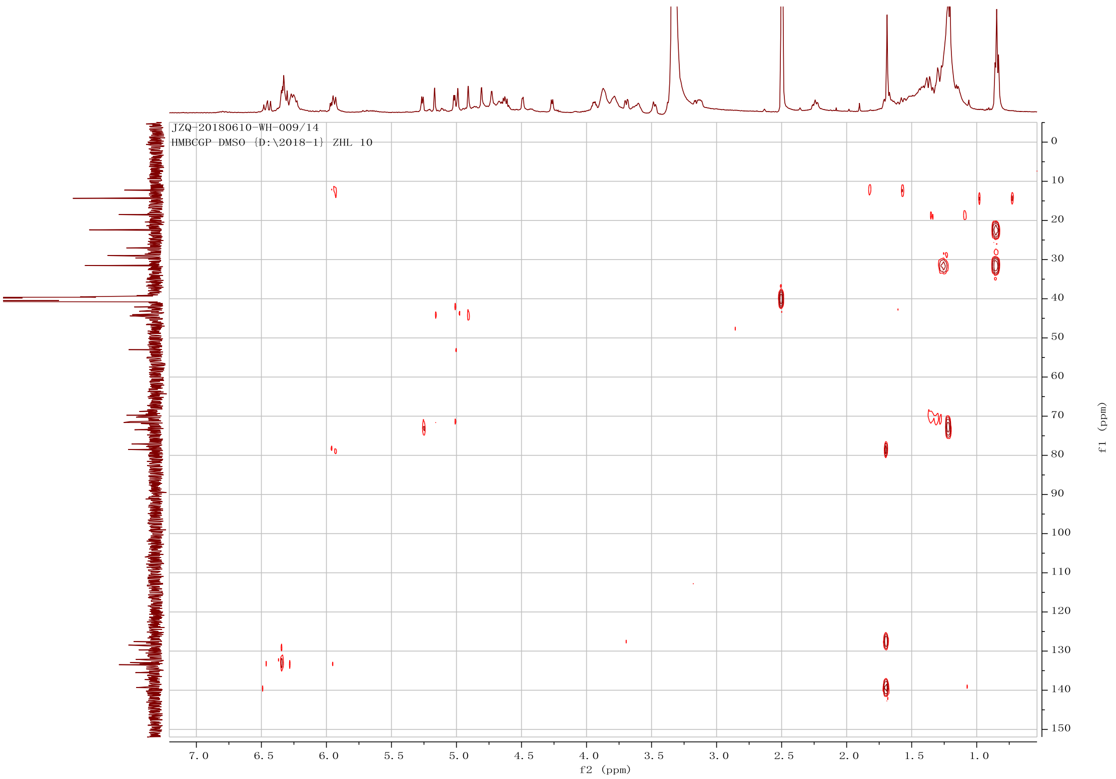

Figure 6. HMBC spectrum of WH02 (600 MHz, DMSO-d_6).

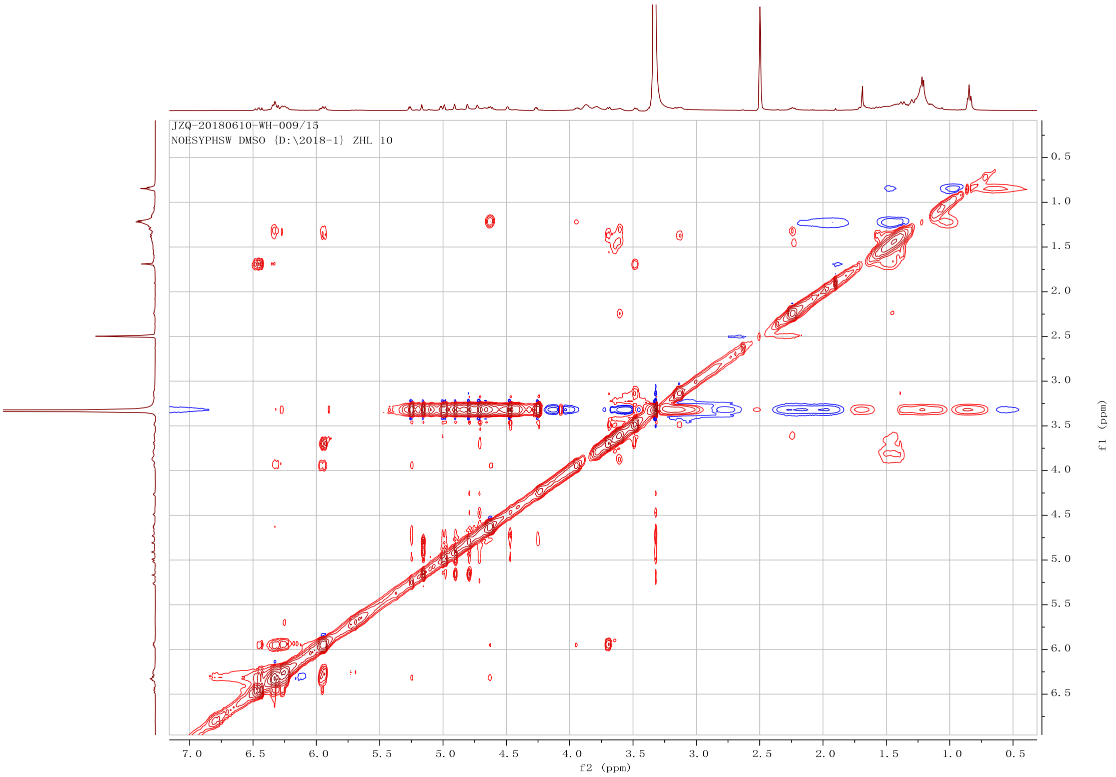

Figure 7. NOESY spectrum of WH02 (600 MHz, DMSO-d_6).

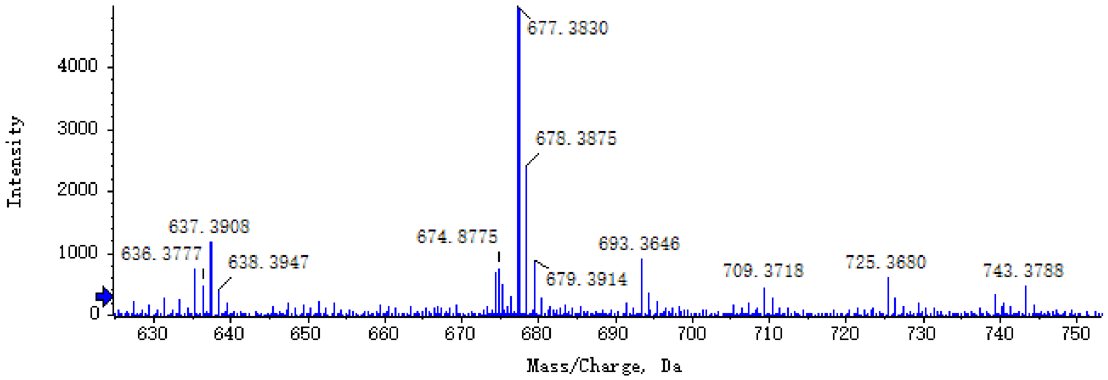

Figure 8. HRMS spectrum of WH02.
